# Characterization of MreCD in *Streptococcus mutans*

**DOI:** 10.1080/20002297.2025.2487643

**Published:** 2025-04-04

**Authors:** Victor Chan, Tessa Holcomb, Justin R. Kaspar, Robert C. Shields

**Affiliations:** a Department of Oral Biology, University of Florida, Gainesville, FL, USA; b Department of Biological Sciences, Arkansas State University, Jonesboro, AR, USA; c Division of Biosciences, Ohio State University, Columbus, OH, USA

**Keywords:** Cell elongation, cell division, microbial interactions, biofilm, *Streptococcus mutans*

## Abstract

**Background:**

Activities that control cell shape and division are critical for the survival of bacteria. However, little is known about the circuitry controlling these processes in the dental caries pathogen *Streptococcus mutans*.

**Methodology:**

We designed experiments to characterize two genes, *mreC* and *mreD*, in *S. mutans.* Assays included cell morphology imaging, protein interaction analysis, transcriptomics, proteomics, and biofilm studies to generate a comprehensive understanding of the role of MreCD in *S. mutans*

**Results:**

Consistent with *mreCD* participating in cell elongation, cells lacking these genes were found to be rounder than wild-type cells. Using bacterial two-hybrid assays, interactions between MreCD and several other proteins implicated in cell elongation were observed. Further characterization, using proteomics, revealed that the surface-associated proteome is different in mutants lacking *mreCD*. Consistent with these changes we observed altered sucrose-mediated biofilm architecture. Loss of *mreCD* also had a noticeable impact on bacteriocin gene expression, which could account in part for the observation that *mreCD* mutants had a diminished capacity to compete with commensal streptococci.

**Conclusion:**

Our results provide evidence that cell elongation proteins are required for normal *S. mutans* physiology and establish a foundation for additional examination of these and related proteins in this organism.

## Introduction

Some members of the mutans group of streptococci colonize the oral cavity of humans and have been implicated as primary etiologic agents of dental caries (tooth decay) [1–3]. The most extensively studied mutans streptococcus is *Streptococcus mutans* [4,5], largely due to its clear relationship to caries pathogenesis and to the ease with which it can be genetically manipulated [5,6]. *S. mutans* is an ovococcus (ovid-shaped) Gram-positive microorganism. The cell envelope, as with other Gram-positives, is defined by the thick layer of peptidoglycan that surrounds the cell membrane. The cell wall also contains polysaccharides that are anchored to the peptidoglycan, including L-rhamnose glucose glycopolymers that are essential for *S. mutans* cell biology and virulence [7,8]. Exactly how the ovococcus shape of *S. mutans* is attained and maintained is not well understood, but likely has parallels with the ovococcus *Streptococcus pneumoniae*, where cell shape is achieved by septal and peripheral modes of cell wall synthesis [9,10]. In *S. pneumoniae*, peripheral cell wall synthesis occurs at the midcell and is coordinated by proteins either known to, or likely to, contribute to cell elongation [10]. These proteins include PBP2b, PBP1a, RodA, RodZ, MltG, MreC and MreD [9,11], along with the more recently identified cell elongation proteins CozE/CozEb [12,13] and EloR/Jag/KhpB [14–16]. Of these, the bifunctional glycosyltransferase‐transpeptidases PBP1a has been studied in *S. mutans* with observed defects in environmental stress tolerance, autolysis and cell morphology [17]. In *S. pneumoniae*, although its primary function is peripheral cell wall synthesis, PBP1a may also contribute to cell division and peptidoglycan maturation [9,18,19]. The functions of other known *S. pneumoniae* cell elongation proteins in *S. mutans* have not been explored.

Our interest in cell elongation proteins originates from an essential gene screen in *S. mutans* using transposon sequencing (Tn-seq) [20]. Insertions within the genes *mreC* and *mreD* were over-represented after selective pressure, which is suggestive that loss of these genes increased fitness. *S. mutans mreCD* (SMU_20 and SMU_21) are organized as an apparent operon that is flanked by tRNA genes and glucan binding protein B (*gbpB*; *pcsB* in *S. pneumoniae* appears to be an orthologue of *gbpB*) [20]. Northern blot analysis has shown that the downstream gene, *gbpB*, is monocistronic and does not share a transcript with *mreCD* [21], consistent with *S. pneumoniae* (where *gbpB* is named *pcsB)* [22]. The functions of MreC and MreD in streptococci are poorly characterized, but they are thought to contribute to peripheral peptidoglycan synthesis, possibly via positioning or regulating the activity of PBPs. Consistent with their participation in cell elongation, depletion of *mreCD* expression in *S. pneumoniae* D39 causes cells to become more spherical [22]. Coccus-shaped streptococci (*e.g. Streptococcus agalactiae* and *Streptococcus pyogenes*) lack these genes, whereas ovococcus-shaped streptococci harbor them, which may again be consistent with a role in cell elongation. However, certain coccus shaped bacteria, such as *Staphylococcus aureus*, do encode homologues of these proteins [23]. There are also key differences and similarities when compared to rod-shaped organisms. For example, both *Escherichia coli* and *Bacillus subtilis* have a *mreBCD* operon with all components of this operon being important for cell shape [24]. Evidence from these rod-shaped bacteria suggests that MreB is a key coordinator of rod-shaped wall construction (with exceptions) and that loss of *mreB* promoted the evolution of coccus-shaped organisms [24]. Determining what the necessary components are for cell-shape determination and how these influence bacterial cell physiology are fundamental questions.

As another measure of the importance of *mreCD*, these genes are essential (lethal if mutated) in certain strains of *S. pneumoniae* and rod-shaped bacteria [22,25,26]. The essentiality of MreCD is strain and species-dependent, *e.g*. not essential in *S. pneumoniae* R6 or *S. aureus* COL [23,27]. Our preliminary studies suggest that *mreCD* are not essential for the viability of *S. mutans* UA159 [20]. Further analysis here confirms this but shows that essentiality may also be strain dependent. We further show that deletion of *mreCD* increases the rate of autolysis and leads to cell rounding. MreCD interact with each other and other putative cell elongation proteins. Cells lacking *mreCD* express lower levels of bacteriocin-related genes and this leads to a reduction in killing of commensal streptococci. Finally, loss of *mreCD* causes changes in the cell surface-associated proteome, which results in altered sucrose-mediated biofilm architecture.

## Materials and methods

### Bacterial strains and growth conditions

*Streptococcus mutans* strains were cultured from single colonies in Brain Heart Infusion (BHI, Difco) broth. Unless otherwise stated *S. mutans* was routinely cultured at 37°C in a 5% CO_2_, microaerophilic atmosphere. *E. coli* strains were routinely cultured in LB broth (Lennox formula; 10 g/L tyrptone, 5 g/L yeast extract and 5 g/L NaCl) at 37°C with aeration. Antibiotics were added to growth media at the following concentrations: kanamycin (1.0 mg/mL for *S. mutans*, 50 µg/mL for *E. coli*), ampicillin (100 µg/mL for *E. coli*). A list of strains and plasmids (Table S3) and oligonucleotide primers (Table S4) can be found in the supplementary material.

### Construction of strains

Standard DNA manipulation techniques were used to engineer plasmids and strains [28]. A PCR ligation mutagenesis method was used to replace genes and non-coding regions with non-polar kanamycin markers [29]. For each gene deletion, primers A and B were designed to amplify 500–600 bp upstream of the coding sequence (with ca. 50-bp overlapping the coding sequence of the gene). Primers C and D were designed to amplify 500 to 600 bp downstream of the coding sequence (with ca. 50-bp overlapping the coding sequence of the gene). Primers B and C contained *Bam*HI restriction enzyme sites for ligation of the AB and CD fragments to a non-polar kanamycin cassette digested from plasmid pALH124 [30]. Transformants were selected on BHI agar containing kanamycin. Double-crossover recombination, without introduction of nearby secondary mutations, was confirmed by PCR and Sanger sequencing using primers E and F, away from the site of recombination.

### Genome sequencing

Genomic DNA was isolated from strains using a MasterPure Gram Positive DNA (Epicentre) purification kit with modifications as previously described [31]. After DNA purification, total DNA concentration and purity were measured using a NanoDrop spectrophotometer (Thermo Fisher Scientific). Five nanograms of DNA from each strain was prepared for next-generation whole-genome shotgun sequencing using the Illumina Nextera-XT library preparation and indexing kit. Libraries were normalized, pooled at a final concentration of 2 nM, and sequenced on an Illumina MiSeq using the Illumina MiSeq v2 kit with paired-end sequencing and 250-bp reads. Reads were demultiplexed using Illumina software. The pipeline A5 [32] was used for genome assembly and the quality of assembled genomes was checked using QUAST [33]. Strain sequences were pairwise aligned against the deposited *S. mutans* UA159 GenBank file (NC_004350.2) using the Mauve multiple genome alignment tool (version 2.4.0) (60), and SNPs unique to that variant from the parental strain were called. SNPs in genes Smu20_03743 and Smu20_06741 were confirmed via Sanger sequencing (Eurofins Genomics, Louisville, KY, USA) (Table S4).

### Scanning electron microscopy

After culturing bacterial strains to an OD_600_ = 0.5, 60 µL of 25% glutaraldehyde (EM grade) was added to each 940 µL of bacterial culture. Samples were then lightly mixed for 1 h, before cells were washed three times with 0.2 M sodium cacodylate buffer (4.28 g sodium cacodylate, 100 mL dH_2_O, pH 7.2). After washing, cells were fixed with 3% glutaraldehyde in cacodylate buffer overnight at 4°C. After fixation, cells were washed a further three times in cacodylate buffer. Fixed samples were placed on polycarbonate membranes (treated with poly-L-lysine) and treated with OsO_4_. Next, samples were dehydrated in a series of steps: 25% EtOH, 50% EtOH, 75% EtOH, and 100% EtOH. After critical point drying, and mounting onto aluminum stubs, samples were sputter coated with gold. Samples were imaged using a Hitachi SU5000 Schottky Field Emission scanning electron microscope.

### Competition assays

For competition assays, strains were cultured in rich media (BHI). An inoculum of 1 × 10^6^ CFUs/mL of wild-type and mutant were added to pre-warmed media and cultured for 24 h in a microaerophilic environment at 37°C. At 0 h, 6 h and 24 h of the experiment bacteria were serially diluted and plated onto BHI and BHI-kanamycin agar. Wild-type and mutant strains were enumerated (wild-type CFU were derived by subtracting mutant CFU from total CFU on BHI agar) and the competitive index was calculated using the following formula: (t_end_ mutant CFU/t_end_ wild-type CFU)/(t_start_ mutant CFU/t_start_ wild-type).

### Autolysis assays

For autolysis assays we followed a previously published protocol [34]. *S. mutans* cells were grown to an OD_600_ = 0.7. Cells were harvested by centrifugation and washed with phosphate-buffered saline. The cells were then resuspended in an autolysis buffer (20 mm potassium phosphate buffer (pH 6.5) containing 1 M KCl, 1 mm CaCl_2_, 1 mm MgCl_2_, and 0.4% sodium azide). Autolysis was then monitored by measuring the OD_600_ of the cell suspension using a Bioscreen C Automated Microbiology Growth Curve Analysis System (cell suspensions were incubated at 44°C).

### Bacterial two-hybrid screens

Bacterial adenylate cyclase two-hybrid (BACTH) assays were performed by following the supplier’s instructions (Euromedex). A full description of this bacterial two-hybrid system can be found in Karimova et al. [35]. Briefly, genes of interest were cloned into the two adenylate cyclase plasmids pUT18C and pKT25 (see Table S4 for oligonucleotide sequences). This cloning was performed in *E. coli* 10-beta. After cloning was validated with Sanger sequencing of the cloned regions, plasmid constructs were transformed into electrocompetent *E. coli* BTH101. For the transformation, both plasmids pUT18C and pKT25 were co-transformed simultaneously. After selecting for co-transformations (LB plus 100 µg/mL ampicillin and 50 µg/mL kanamycin), colonies were grown overnight in LB media. The following day 5 µl of each interaction strain was plated onto M9 agar containing glucose and 50 µg/mL 5-Bromo-4-chloro-3-indolyl-β-D-galactopyranoside (X-gal). Positive and negative protein interaction controls were also plated. Agar plates were incubated at 30°C for 16 h overnight and imaged. Transformations were done in triplicate with selected images representative of three biological replicates.

### Proteomics

*Tandem mass tag mass spectrometry. S. mutans* wild-type and ∆*mreCD* mutants were cultured to mid-log (~0.4–0.6 OD_600_) and cell pellets were collected. For each strain, a total of five biological replicates were collected. Cell pellets were sent on dry ice to the IDeA National Resource for Quantitative Proteomics at the University of Arkansas for Medical Sciences (UAMS). Proteins were extracted using chloroform/methanol and digested with trypsin. Next, the peptides were labeled and then fractionated using high-performance liquid chromatography (HPLC). LC-MS was completed using an Orbitrap Eclipse (Thermo Scientific) platform. The quantitative proteomics software, MaxQuant, was used to search for *S. mutans* UA159 proteins, and QC, normalization, and differential expression analysis were completed afterward.

*Two-dimensional differential gel electrophoresis*. Cell surface-associated proteins were extracted using mutanolysin treatment as previously described [36]. Proteins were precipitated using TCA/acetone. First, one volume of 20% TCA was added to cell surface-associated protein supernates, with cooling on ice for 1 h. Next, four volumes of 100% cold acetone were added to the protein solution, mixed, and placed in a −20°C freezer overnight. Precipitated proteins were collected by centrifugation and washed with ice cold 80% acetone. The protein pellet was sent to Applied Biomics for two-dimensional differential gel electrophoresis (2D-DIGE). Protein spots were picked and analyzed by LC-MS/MS to allow for their identification.

### Biofilm assays and imaging

For crystal violet assays, cells were cultured to an OD_600_ = 0.5. Next, cells were washed and transferred 1:50 into microtiter wells (96-well polystyrene plates) containing 200 µL FMC media with either 25 mm glucose or 12.5 mm sucrose as the carbohydrate source. Biofilms were cultured for 24 h, washed, and stained with crystal violet (0.1% w/v). Acetic acid (7%) was used to solubilize crystal violet before reading A_570_ on a Synergy HT microplate reader. For microscopy, biofilms were cultured on glass coverslips as previously described [31]. Biofilms were stained with the LIVE/DEAD *Bac*Light Bacterial Viability kit. Biofilms were examined using a spinning disk confocal system connected to a Leica DMIRB inverted fluorescence microscope equipped with a Photometrics cascade-cooled EMCCD camera. SYTO 9 fluorescence was detected by excitation at 488 nm and emission was collected using a 525 nm (±25 nm) bandpass filter. Propidium iodide fluorescence was detected using a 642-nm excitation laser and a 695-nm (±53 nm) bandpass filter. All images were collected using a 63X/1.40 oil objective lens. Laser power (70%) and exposure time (250 ms) were kept constant for all images. Image acquisition and processing was performed using VoxCell (VisiTech International) and Imaris (Bitlane). Images are representative of three independent experiments and during each experiment at least three micrographs were taken per strain.

### RNA sequencing

RNA-sequencing was performed as described previously by Zeng et al. [37]. RNA was extracted from OD_600_ = 0.5 bacterial cultures using the RNeasy Mini Kit. Next, RNA was treated with the MICROB*Express* Bacterial mRNA Enrichment Kit (ThermoFisher Scientific) to remove 16S and 23S rRNAs. After this step the quality of mRNA was assessed using an Agilent Bioanalzyer (Agilent Technologies) at the University of Florida NGS core facility. Following the quality check, cDNA libraries were made using the NEBNext Ultra II Directional RNA Library Prep Kit and NEBNext Multiplex Oligos for Illumina (New England Biolabs). Deep sequencing was performed by the University of Florida NGS core facility on an Illumina NextSeq500 DNA sequencing machine. After sequencing, read counts were aligned to the *S. mutans* UA159 genome using bioinformatics tools hosted on the Galaxy server maintained by the Research Computing Center at the University of Florida. Gene expression changes between samples were quantified with Degust (http://degust.erc.monash.edu/) using the edgeR methodology.

### Microbial antagonism assays

Wild-type and mutant strains of *S. mutans* were stabbed into BHI agar, using a toothpick, and left to incubate for 24 h in a 5% CO_2_ environment at 37°C. Next, 10 mL soft BHI agar (0.75% agarose) was inoculated with 10 μL *S. gordonii* DL1 or *S. sanguinis* SK150 overnight culture. The soft agar bacterial indicator strain mix was then overlaid onto the BHI agar plates with stabbed *S. mutans* colonies. After 24 h plates were imaged, and zones of clearance were calculated using ImageJ image analysis software.

### Statistical analysis

Three biological replicates were conducted for each experiment. We calculated means and standard errors to assess baseline differences. To test for significant differences, we performed a Student’s t test to compare the means of two groups, and if the *p* value was less than 0.05 we considered the difference in means statistically significant.

### Data availability

Genome sequencing is available to download from the NCBI under the BioProject accession number PRJNA700622. The original RNA-seq data from this study was uploaded to the GEO database (https://www.ncbi.nlm.nih.gov/geo/) with the accession number GSE165679.

## Results

### *mreCD* are not essential in *S.* *mutans* strain UA159

To determine whether *mreCD* were absolutely required for the survival of *S. mutans*, as is the case for *S. pneumoniae* D39 [22], we replaced *mreC*, *mreD* and *mreCD* of *S. mutans* UA159 with a non-polar kanamycin resistance cassette. As shown in Figure S1, transformants of UA159 were readily obtained with the *mre-*mutating DNAs, with the number of transformants being similar to that obtained with transformation of plasmid pIB184 carrying an erythromycin resistance gene (Figure S1). All ∆*mreC*, ∆*mreD*, and ∆*mreCD* strains examined grew normally in BHI broth (Figure S2).

The essentiality of *mreCD* is strain dependent in *S. pneumoniae* [22]. With this in mind, we introduced the *mreCD* mutant DNA constructs into two genomically distinct *S. mutans* isolates, Smu20 and Smu93 [38]. Although not extensively tested, we anticipate that these strains have different cell-surface characteristics compared to UA159. For example, there are significant differences in the capabilities of these two strains to attach to surfaces and form biofilms. Smu20 forms biofilms poorly in the presence of sucrose, apparently due to a recombination event between the *gtfB* and *gtfC* genes [38]. Surprisingly, this strain forms robust biofilms in glucose-containing media, comparable to *S. mutans* UA159 biofilms formed in sucrose. Strain Smu93 forms a biofilm equally as well in both glucose and sucrose [38]. *S. mutans* strains are grouped according to four serotypes (*c*, *e*, *f*, and *k*) differentiated by differences in the chemical composition of the cell envelope. *S. mutans* Smu20 is a serotype *c* strain (like UA159) and *S. mutans* Smu93 is a serotype *e* strain. We reasoned that differences in gene content/cell envelope composition could change the dispensability of genes like *mreCD*. While ∆*mreCD* transformed with high efficiency into Smu93, very few colonies were obtained when Smu20 was transformed with the ∆*mreCD* DNA (Figure S1). Smu20, however, was highly transformable, as evidenced by efficient transformation with pIB184 (Figure S1). We picked three ∆*mreCD*_Smu20_ strains for further analysis, as the low transformability could indicate that suppressor mutations were necessary to offset lethality of *mreCD* inactivation. When grown in BHI broth, ∆*mreCD*_Smu20_ strains grew as well or better than the wild-type Smu20 (Figure S2); although cell shape defects may preclude accurate comparisons of growth efficiency by optical density. Noticeably, the growth profiles of the three independently selected ∆*mreCD*_Smu20_ strains were different from each other. ∆*mreCD*_Smu93_ displayed a similar lag phase and indistinguishable exponential phase growth rates when compared with the parental strain (Figure S2).

Loss of essential genes can be compensated for by suppressor mutations elsewhere in the genome. To investigate if this happened in any of the ∆*mreCD* mutant strains, we extracted chromosomal DNA and determined the genome sequences with next-generation sequencing technologies. No single-nucleotide polymorphisms (SNPs), or other genetic changes, were observed in the ∆*mreCD*_UA159_ or ∆*mreCD*_Smu93_ mutant strains. However, two SNPs were present in ∆*mreCD*_Smu20_, both causing nonsynonymous mutations. The presence of the SNPs in Smu20_03743 (AAT>AAG; N261K) and Smu20_06741 (AGC>ATC; S59L) was confirmed by PCR and sequencing. Smu20_03743 is a predicted hydrolase (HAD superfamily) and Smu20_06741 is annotated as a LytR-family response regulator. Figure S3 shows the location of each nonsynonymous mutation within the two proteins. The function of the hydrolase protein is unknown. A homologous *lytR* gene in UA159 (*lytT*, SMu.576) regulates autolysis and affects the extent of chaining of cells [39]. Immediately downstream of the *lytRS* two component system is the *lrgAB* system, which has functions related to autolysis and pyruvate transport [40,41].

### Over-representation of *mreC* and *mreD* transposon mutants during Tn-seq is related to a lytic phenotype

**Figure 1.**
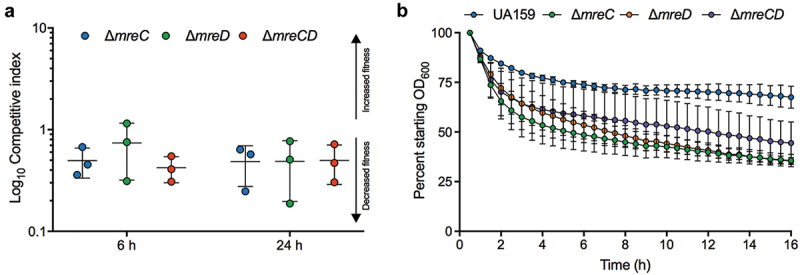
Impact of *mreC*, *mreD* or *mreCD* deletion on cell fitness and autolysis. (a) Competitive index (CI) values reflect altered capacity of mutants to compete with *S. mutans* UA159. Strains were mixed 1:1 in rich media and colony forming units of competing strains were enumerated at inoculation, then after 6 h and 24 h of incubation. Horizontal bars indicate mean CI values. (b) The autolytic activities of strains were measured using a Bioscreen C system. Cells were incubated in an autolysis buffer (see materials and methods) at 44°C to promote cell lysis. Error bars represent standard error from three biological replicates (on different days).

We first became interested in *mreCD* when our prior Tn-seq essential gene studies revealed that insertions in these genes were over-represented after culture in rich and defined media [20]. We reasoned that there could be two possible explanations for this finding: loss of *mreC* or *mreD* increased the fitness of *S. mutans* or mutations in these genes yielded cells that were more easily lysed in our DNA isolation protocol; the latter increasing the representation of genomic DNA extracted from the mutants in the sequencing libraries. Loss of *mreCD* in both *B. subtilis* 168 and *S. pneumoniae* D39 increases cell lysis, although the genes are essential in these organisms [22,25]. To determine why *mreCD* were overrepresented in Tn-Seq, we began by measuring changes in the competitive fitness of *mreCD* mutants in the *S. mutans* UA159 genetic background. For competition assays, equal amounts of *S. mutans* UA159 and *mreCD* mutants were inoculated into rich medium and the relative proportions of these strains were measured at time 0, then at 6 and 24 hours of incubation. At both the 6 and 24-hour time-points the ∆*mreC*, ∆*mreD* and ∆*mreCD* mutants were slightly outcompeted by the wild-type strain (Figure 1(a)). This outcome suggests that loss of *mreCD* does not increase the fitness of *S. mutans*, although co-culture competitions are distinct from Tn-seq competitions, which contain thousands of different mutants. Next, we investigated how loss of these genes impacted autolysis. For these assays, strains were cultured until they were close to late exponential phase, washed and re-suspended in an autolysis buffer [34]. This assay showed that ∆*mreC*, ∆*mreD* and ∆*mreCD* were more autolytic than wild-type *S. mutans* (Figure 1(b)). The increase in the rate of autolysis varied by strain with ∆*mreCD* showing a decreased rate of autolysis, compared to the ∆*mreC* and ∆*mreD* single mutants. We therefore cannot exclude the possibility that the observed changes in autolysis contributed to the increased number of transposon insertions measured in *mreCD* by Tn-seq. The important point here, though, is that disruption of *mreC, mreD* or both genes increased autolysis.

### MreCD are necessary for normal cell width and length

**Figure 2.**
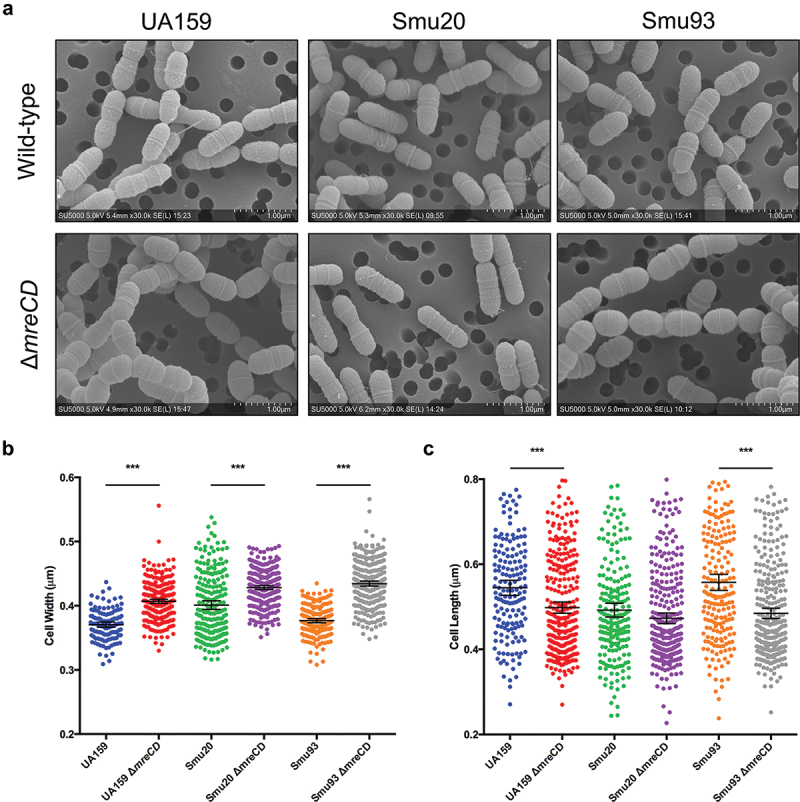
Deletion of *mreCD* leads to more spherical bacterial cells. (a) SEM (scanning electron microscopy) images of *S. mutans* wild-type and ∆*mreCD* strains. Cells of *S. mutans* were grown in rich media to an OD_600_ = 0.5 before fixing and preparing for SEM. Scale bar = 1 µm. Bacterial cell widths (b) and lengths (c) were measured using ImageJ software from hundreds of cells visualized with SEM. Error bars indicate standard error with the middle horizontal bar indicating the mean. Statistically significant differences are indicated based on the Student’s t test (***p < 0.001).

MreCD are required for normal cell shape and cell elongation in ovococcus and rod-shaped bacteria [22,25]. To test if MreCD have a similar role in *S. mutans*, we analyzed wild-type and mutant strains with scanning electron microscopy (SEM). By SEM, there were no major differences in the appearance of the cell surfaces of wild-type and ∆*mreCD* strains (Figure 2(a)). The shape of ∆*mreCD* strains was symmetrical and there were no obvious division septum defects (Figure 2(a)). To compare cell dimensions between wild-type and ∆*mreCD* strains, we used ImageJ to measure cell widths and lengths. The cell widths of all three strains (UA159, Smu20 and Smu93) were significantly (*p* > 0.001) increased when *mreCD* was deleted in these backgrounds (Figure 2(a)). Furthermore, the cell lengths of both UA159 and Smu93 were significantly (*p* > 0.001) less in strains lacking *mreCD* (Figure 2(c)).

### MreC and MreD interact with putative elongasome proteins

**Figure 3.**
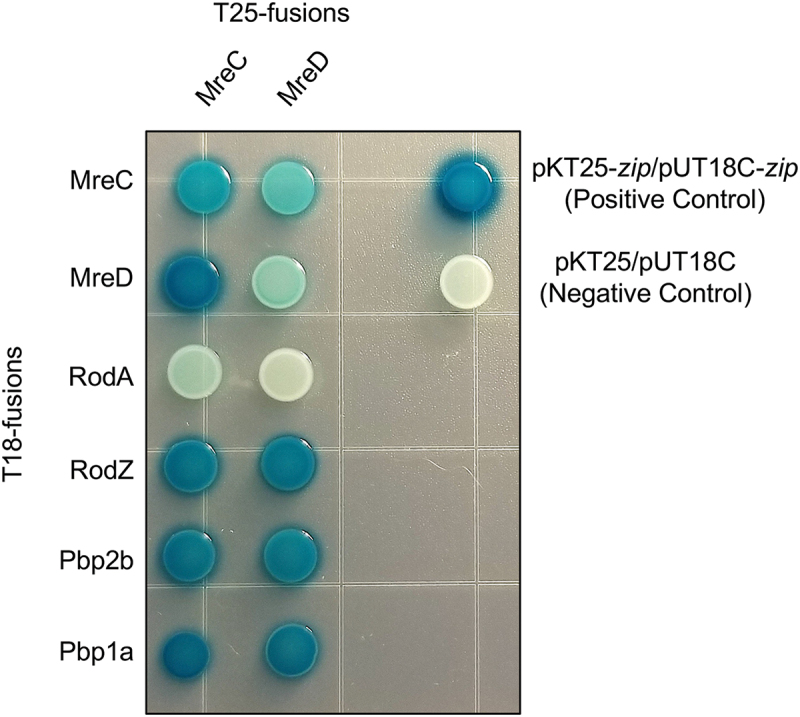
MreC and MreD interact with each other and other putative cell elongation proteins. Cell elongation proteins were cloned and fused with adenylate cyclase fragments T18 and T25 and co-expressed in an *E. coli cya*^−^ strain. Protein interactions restore adenylate cyclase activity resulting in synthesis of cAMP followed by cap-activated expression of β-galactosidase. Strains were spotted on M9-glucose agar and incubated at 30°C for 24 h. A blue spot colony indicates a positive interaction between proteins whereas a colorless colony indicates no interaction. Positive and negative controls (supplied by Euromedex) are shown. Images are representative of at least three independent biological replicates.

Although the exact functions of MreC and MreD are still poorly understood, they can interact with several other proteins that contribute to bacterial cell elongation [14,42,43]. It has been proposed that by interacting with PBP2 and RodA in *E. coli*, MreC and MreD regulate the activity of these cell wall synthesis enzymes [42]. To investigate if MreC and MreD interact with cell elongation proteins in *S. mutans*, we used a bacterial two-hybrid (B2H) system. With the particular B2H system used, if two proteins of interest interact, the catalytic domain of adenylate cyclase is restored, allowing expression of a reporter gene activity: in this case *lacZ*. The following putative *S. mutans* elongasome proteins were cloned into B2H plasmids: *mreC*, *mreD*, *rodA* (SMu.1279c), *rodZ* (SMu.2152c), *pbp2b* (SMu.597), and *pbp1a* (SMu.467). Recently, another member of the cell elongation protein complex was identified in *S. pneumoniae*, *cozE* (coordinator of zonal elongation) [12,13]. It appears that there might be multiple copies of the *cozE* gene in *S. mutans*, as is the case in *Staphylococcus aureus* and *S. pneumoniae* [44,45], so we did not include *cozE* genes in our B2H screen. After plasmid cloning, two plasmids, each containing a cell elongation protein gene and a fragment of adenylate cyclase, were transformed into *E. coli* BTH101. Interactions were probed by screening for β-galactosidase-producing colonies on M9 minimal media agar containing X-gal. As shown in Figure 3, recombinant MreC and MreD interacted with most of the cell elongation proteins we tested. The only plasmid combinations for which we did not observe significant *lacZ* activity were MreD with itself and MreC or MreD with RodA.

### Loss of *mreCD* modifies the transcriptome and proteome of *S.* *mutans*

**Figure 4.**
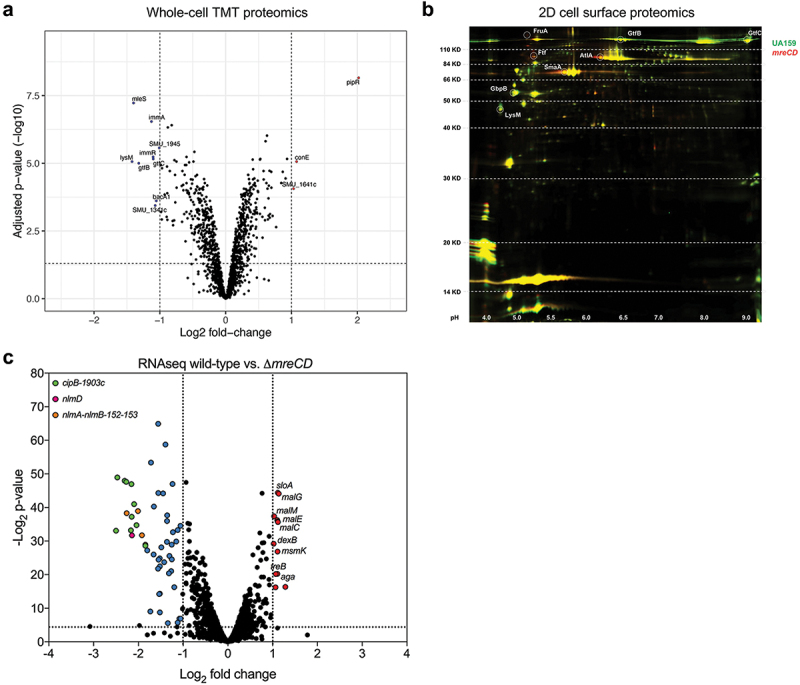
The proteome and transcriptome of *mreCD* mutants differ from wild-type *S. mutans*. (a) Volcano plot of whole-cell proteomics showing protein expression values for ∆*mreCD* relative to wild-type *S. mutans* cultured in rich media (see materials and methods for description of data analysis). Differentially expressed proteins (log2 fold-change >1 or <-1; adjusted p-value ≤0.05) are highlighted in red (up-regulated) and blue (down-regulated). (b) A representative image of a 2D-DIGE experiment performed with cell surface-associated protein extracted from wild-type and ∆*mreCD S. mutans* strains. *S. mutans* UA159 protein samples were labelled with Cy3 (green color) and *S. mutans* ∆*mreCD* protein samples were labelled with Cy5 (red color). Protein spots (circles) were identified with mass spectrometry and were picked because of differential expression between samples. Proteins more strongly expressed in ∆*mreCD* fluoresce red (*e.g*. AtlA) whereas proteins more strongly expressed in UA159 fluoresce green (*e.g*. GtfB). (c) Volcano plot of rna-seq transcriptome data showing gene expression values for ∆*mreCD* relative to wild-type *S. mutans* cultured in rich media (see materials and methods for description of data analysis). Differentially expressed genes (FDR ≤0.01) are highlighted in red (up-regulated) and blue (down-regulated). Bacteriocin genes are also highlighted in individual colors with a legend on the graph. Black dotted lines represent boundaries for significantly up- or down-regulated genes.

To assess the global importance of MreCD we conducted whole-cell proteomics, cell-surface-proteomics, and transcriptomics. We began by using tandem mass tag (TMT) quantitative proteomics to compare proteins between wild-type and ∆*mreCD* cell lysates. A total of 1,510 proteins were detected from *S. mutans* cell lysates. Of these, 696 were significantly up- or down-regulated (adjusted *p* value < 0.05) (Figure 4(a)). Of these, nine proteins were downregulated by < −1, and three by > 1 (Table S1). The down-regulated proteins were SMU_1341c, SMU_1945, SMU_2147c, MleS, GtfB, GtfC, ImmR, ImmA and BacA1. The up-regulated proteins were SMU_198c, SMU_1641c, and SMU_2134.

Next, we analyzed the protein expression profiles of UA159 and ∆*mreCD* by two-dimensional differential gel electrophoresis (2D-DIGE). Cell-surface associated proteins were extracted using a protocol developed by McNab and Jenkinson [36]. Proteins were then labelled with CyDye DIGE fluors, and the two samples were simultaneously separated on a single 2D gel (Figure 4(b)). Comparative analysis quantified protein expression ratios (PER) between the two samples. Protein spots were chosen based on a PER > 1.5. A total of 24 spots were then analyzed by LC-MS/MS, and the PER of these spots is described in Table 1. Proteins with decreased abundance in ∆*mreCD* included GtfB, GtfC and GbpB, whereas FruA, Ftf, AtlA and SmaA were increased in the ∆*mreCD* mutant, compared to wild-type *S. mutans*.

**Table 1.** 2D-DIGE.

Protein Name	Product	Protein MW	Protein pI	Protein Score C.I.%	Peptide Count	Fold-change
GtfB	Glucosyltransferase-I	165,745	6.48	100	40	−2.1
GtfC	Glucosyltransferase-SI	162,886	9.10	100	25	−1.6
FruA	Fructan hydrolase	158,563	5.03	100	41	2.1
AtlA	Autolysin	107,128	5.52	100	25	2.5
Ftf	Fructosyltransferase	87,331	5.13	100	9	1.8
SmaA	Putative peptidoglycan hydrolase	67,417	5.66	100	28	2.2
GbpB	Putative peptidoglycan hydrolase	44,594	4.83	100	9	−1.9
LysM	Peptidoglycan-binding LysM domain	30,453	4.31	100	6	−1.5

To quantify changes in gene expression, we conducted RNA-seq comparing the transcriptomes of wild-type *S. mutans* with ∆*mreCD* cultured in rich medium to OD_600_ = 0.5. Genes with significant changes in expression were defined as those with a log_2_ fold change > 1.0 and a false discovery rate (FDR) < 0.01. A total of 48 genes were down-regulated and 12 genes were up-regulated in the ∆*mreCD* strain (Table S2). Genes related to bacteriocin production (e.g. *cipB*, *nlmD*, *nlmAB*), the putative integrative conjugative element TnSmu1 and late competence (e.g. *comFA*, *comG*, *comEA*) were down-regulated (Figure 4(c)). Several genes involved in carbohydrate utilization were up regulated in the ∆*mreCD* strain, including those associated with catabolism of maltose and trehalose, as well as the multiple sugar metabolism system (msm) (Figure 4(c)). Overall, the changes in the *S. mutans* transcriptome in cells lacking MreCD were modest. There was no significant overlap between the changes in gene expression and the changes in the surface-associated proteome, suggesting that the latter were likely associated with altered secretion or anchoring to the cell surface.

### MreCD are required for optimal bacteriocin production

**Figure 5.**
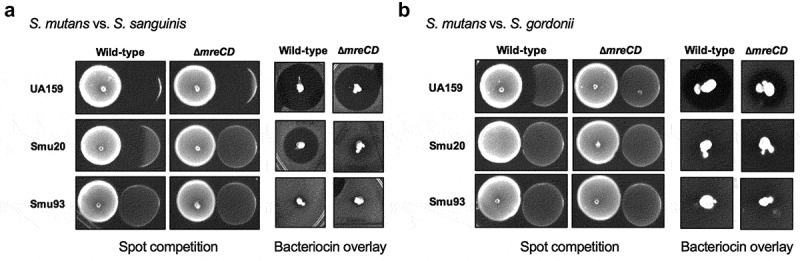
Deletion of *mreCD* modifies the microbial antagonism capabilities of *S. mutans*. Agar-based spot competition and bacteriocin overlay assays between *S. mutans* strains and (a) *S. sanguinis* or (b) *S. gordonii*. For spot competition assays *S. mutans* strains were incubated on the agar for 24 h before commensal strains were spotted, then plates were incubated for a further 24 h. Bacteriocin overlay assays were performed by stabbing *S. mutans* strains into agar and the following day overlaying a soft agar containing commensal bacteria. Plates were incubated for a further 24 h. A zone of clearance close to the *S. mutans* colony suggests that the growth of commensal bacteria was inhibited.

Bacteriocin-related genes were significantly down-regulated in the ∆*mreCD* strain. To explore these results in greater detail, we conducted functional assays for bacteriocin production using both spot competition and bacteriocin overlay assays (Figure 5). For both assays, *S. mutans* strains were inoculated first for 24 hours, then the following day bacteriocin-susceptible strains (*S. gordonii* or *S. sanguinis*) were either overlaid or spotted next to *S. mutans* strains. In agreement with the RNA-seq results, *S. mutans* UA159 was able to compete against *S. gordonii* and *S. sanguinis* better than its ∆*mreCD* mutant. This phenotype extended to the clinical isolate Smu20 when competed against *S. sanguinis*. Neither the wild-type Smu93 or its *mreCD* mutant derivative had any effect on the growth of *S. gordonii* or *S. sanguinis*; Smu93 has a truncated competence stimulating peptide (CSP) that is needed for regulation of bacteriocins [38].

### Alterations in biofilm architecture in *mreCD* mutants

**Figure 6.**
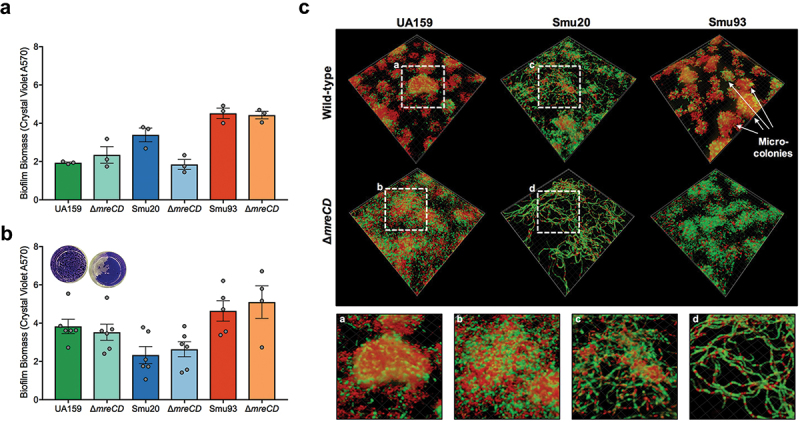
Impact of *mreCD* deletion on sucrose-mediated biofilm architecture. Biofilm biomass was quantified using a crystal violet assay for *S. mutans* strains grown in microtiter wells. Cells were grown in FMC containing glucose (a) or sucrose (b) to assay for sucrose-independent and sucrose-dependent biofilm characteristics respectively. Grey circles indicate individual crystal violet measurements (biological replicates) with the height of the bar indicating mean values; error bars indicate standard error. Pictures from *S. mutans* UA159 and ∆*mreCD* micotiter biofilm accumulation are shown inset into (b). Microcolonies are visible for wild-type *S. mutans* whereas ∆*mreCD* has a smoother biofilm architecture. (c) Sucrose-mediated biofilm formation was also visualized with confocal laser scanning microscopy (CLSM). Biofilms were stained with LIVE/DEAD, with green signifying live cells and red indicating dead cells. Biofilm Z-stack images were processed into three-dimensional images using Imaris software. Magnified parts of images from UA159, Smu20 and ∆*mreCD* mutants are incorporated into the figure. Images are representative of at least three biological replicates and were collected with 63X magnification.

Given that loss of *mreCD* caused changes in surface-associated proteins and autolysis, we next explored if there was any impact on biofilm attachment/accumulation. We grew *S. mutans* wild-type strains and *mreCD* mutant derivatives in polystyrene microtiter wells in the defined medium FMC with either glucose or sucrose as the sole carbohydrate source. After 24 h of incubation, no significant changes in biofilm biomass were observed between strains growing on glucose or sucrose (Figure 6(a,b)). However, there was a noticeable macroscopic change in the appearance of the UA159-derived ∆*mreCD* mutant biofilms in the microtiter wells after crystal violet straining (Figure 6(b) inset). To explore this further, we grew biofilms in FMC-sucrose and stained the bacterial cells with propidium iodide and Syto9. The biofilms were then imaged with confocal laser scanning microscopy and the images were processed with Imaris to create three-dimensional reconstructions. For both *S. mutans* UA159 and Smu93, the wild-type strains formed microcolonies that were typical of those formed by *S. mutans* in the presence of sucrose. For Smu20, cells sparsely coated the surface and no micro-colonies were visible, potentially related to a recombination event between *gtfB* and *gtfC* [38]. Consistent with the appearance of biofilms after crystal violet staining, loss of *mreCD* in the UA159 background altered the ability of cells to form microcolonies (Figure 6(c)). Deletion of *mreCD* in the Smu20 background did not greatly alter the appearance of biofilms, except that there was an increase in cell chaining, a phenotype that was not apparent in the planktonically cultured cells used for SEM (Figure 6(c)). Loss of *mreCD* in the Smu93 background did not appear to appreciably alter biofilm architecture. Taken together, loss of *mreCD* does not reduce biofilm biomass, but can alter the architecture of biofilms in a strain-dependent manner.

## Discussion

MreC and MreD are required for cell elongation in some rod- and ovococcus-shaped bacteria [22,46]. Certain species and strains of bacteria are not viable or are more susceptible to lysis when MreCD are depleted [22,25]. However, the essentiality of MreCD is strain-dependent and, for example, deletion of *mreCD* in *S. aureus* has very little discernable effect [23]. Here, we report the first characterization of *mreCD* in *S. mutans*, including microscopic, proteomic, transcriptomic and phenotypic analyses. Our data provide further support for a role for MreCD in cell elongation of ovococcus-shaped bacteria. The data also demonstrate that cells lacking these proteins are defective in bacteriocin production and that the surface-associated proteome is altered enough to elicit changes in biofilm architecture.

We confirmed the lack of essentiality of MreCD in UA159 by genome sequencing, which showed that the strain had not developed any compensatory mutations. In contrast, deletion of *mreCD* in Smu20, a serotype *c* clinical isolate from a caries-free individual, was more challenging and SNPs were detected in the viable transformants. In *S. pneumoniae*, differences in *mreCD* essentiality between strains have been attributed to changes in the amino acid composition of PBP1a [22]. Moreover, deletion of *pbp1a* in *S. pneumoniae* D39 alleviates *mreCD* essentiality in this strain [12,22]. No SNPs were detected in the *pbp1a* sequence of Smu20-derived ∆*mreCD* strain, but SNPs were present in genes for an uncharacterized hydrolase protein and for a response regulator that controls autolytic behavior, *lytR*. LytR, as part of a two-component system, regulates the expression of *lrgAB* in *S. mutans* UA159 [40,47,48] and *S. aureus* [49]. Although the exact function of the Lrg system is to be determined, it is linked to cell death and lysis decisions in bacteria [50]. Lrg also appears to be involved in pyruvate excretion and uptake, and thus has a central role in cellular physiology in *S. mutans* [41]. It remains to be verified if the SNP within *lytR* is functionally related to suppressing *mreCD* essentiality in Smu20.

Our detection of interactions between MreC with MreD and MreC with PBP1a, PBP2b, and RodZ is consistent with observations in *S. pneumoniae* [12,14]. Although a definitive function for MreC and MreD is lacking in streptococci, the fact that these proteins interact with peptidoglycan synthesis enzymes provides support for the hypothesis that they have the potential to directly affect the cell elongation process. MreCD functioning in this manner would be consistent with the cell rounding phenotype observed in the *mreCD* mutants of *S. mutans* (Figure 3) and other bacteria [22,46], as well as with the observation that perturbations to the cell elongation machinery affect cell lysis [22,25].

During normal cell division, there is a balance between cell wall synthesis (PBP activity) and degradation (hydrolase activity). Loss of *mreCD* appears to disrupt this balance, both in *S. mutans* and in *S. pneumoniae* and *B. subtilis* [22,25]. Several enzymes capable of hydrolyzing peptidoglycan were differently regulated (at the protein level) after the loss of *mreCD*. We observed increased levels of the autolysin enzyme AtlA [51] and the putative murein hydrolase SmaA [52]. In contrast, there were decreased levels of the putative murein hydrolase GbpB. There is significant overlap between the differentially regulated proteins in ∆*mreCD*, including peptidoglycan hydrolases, and the VicRK signal transduction system [53–55]. A regulatory pathway connecting VicRK to cell elongation via MreCD (or other elongation proteins) is conceivable and could be explored in future studies.

It is unknown how a change to the smooth biofilm architecture seen for the MreCD-deficient strains from well-defined microcolonies observed with wild-type UA159 and Smu93 might impact how efficiently *S. mutans* colonizes the oral cavity or causes disease. Sucrose-dependent microcolony formation is linked to Gtf levels (lower in ∆*mreCD*_UA159_) and could be responsible for promoting acidic microenvironments within biofilms [56,57]. Competition within biofilms is another important attribute for successful oral cavity colonization by *S. mutans*, and loss of *mreCD* impacted bacteriocin production. We previously detected changes in bacteriocin production after loss of other proteins involved in regulation and synthesis of the cell envelope [58]. Here, it is likely that changes in the cell envelope alter the peptide signaling dynamics that are required for bacteriocin production [59].

To conclude, we have found that *mreCD* contribute to cell shape, microbial antagonism and biofilm architecture in *S. mutans*. It remains to be determined how MreCD affects peptidoglycan synthesis during cell elongation, both in *S. mutans* and other ovococci. The next step will be determining how MreCD interactions with other putative cell elongation proteins impacts their localization and/or activity, and if the phenotypes displayed by ∆*mreCD* are conserved across these proteins. Further characterization of *S. mutans* cell wall biogenesis is important for revealing novel strategies for disrupting *S. mutans* colonization of or persistence in the oral cavity.

## Supplementary Material

MreCD_SupplementalMaterial.docx
