# Supplementary material for: Characterization of MreCD in Streptococcus mutans
Source: J Oral Microbiol. 2025 Apr 4;17(1):2487643. doi: 10.1080/20002297.2025.2487643 (PMC11980242; doi:10.1080/20002297.2025.2487643)
Supplement: MreCD_SupplementalMaterial.docx [file ZJOM_A_2487643_SM4401.docx]

**Supplemental Material for:**

**Title: Characterization of MreCD in *Streptococcus mutans***

**Authors**: ^1^Victor Chan, ^2^Tessa Holcomb, ^3^Justin R. Kaspar, ^1,2^Robert C. Shields^*^

**Affiliations:**

^1^Department of Oral Biology, University of Florida, Gainesville, Florida, USA

^2^Department of Biological Sciences, Arkansas State University, Jonesboro, Arkansas, USA

^3^Division of Biosciences, Ohio State University, Columbus, Ohio, USA

**This file includes:**

Figs. S1 – S3

Table S1 – S4


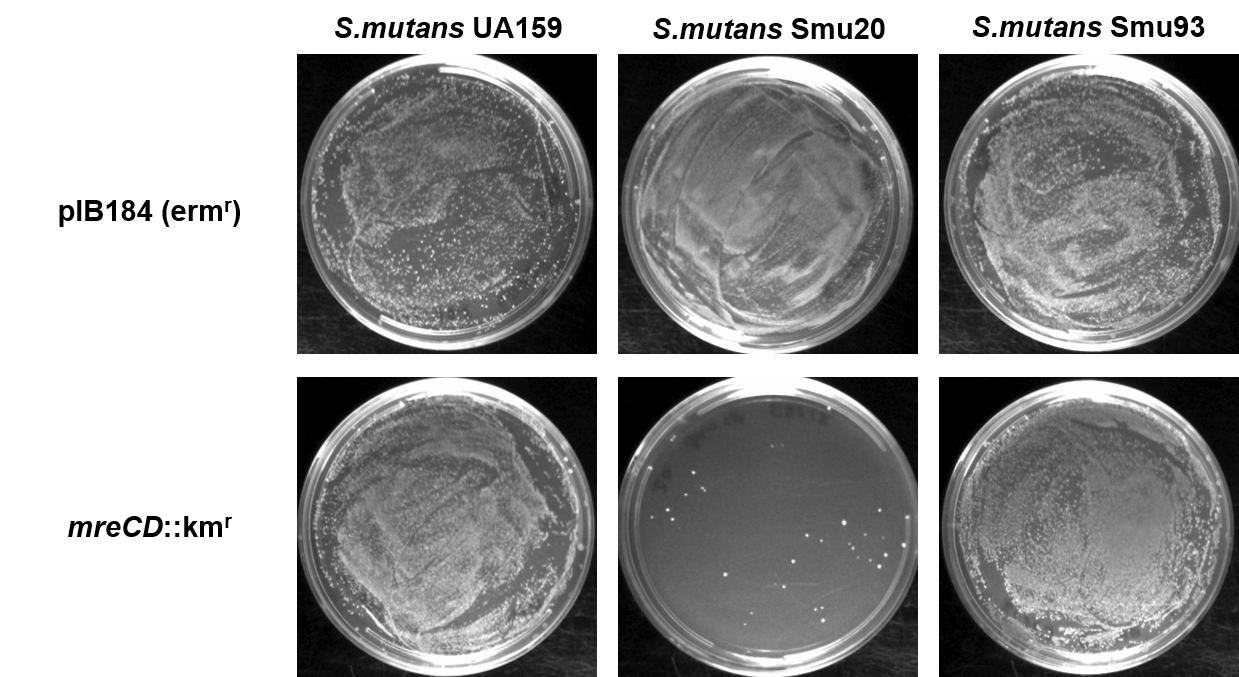


**Figure S1. Transformation efficiency of *mreCD*::km^R^ into *S. mutans* UA159, Smu20 and Smu93.** Top panel shows transformation efficiency of the pIB184 plasmid (erythromycin resistance) used as a control. Bottom panel shows transformation efficiency with *mreCD*::km^R^ as the exogenous DNA added to competent *S. mutans*.

**
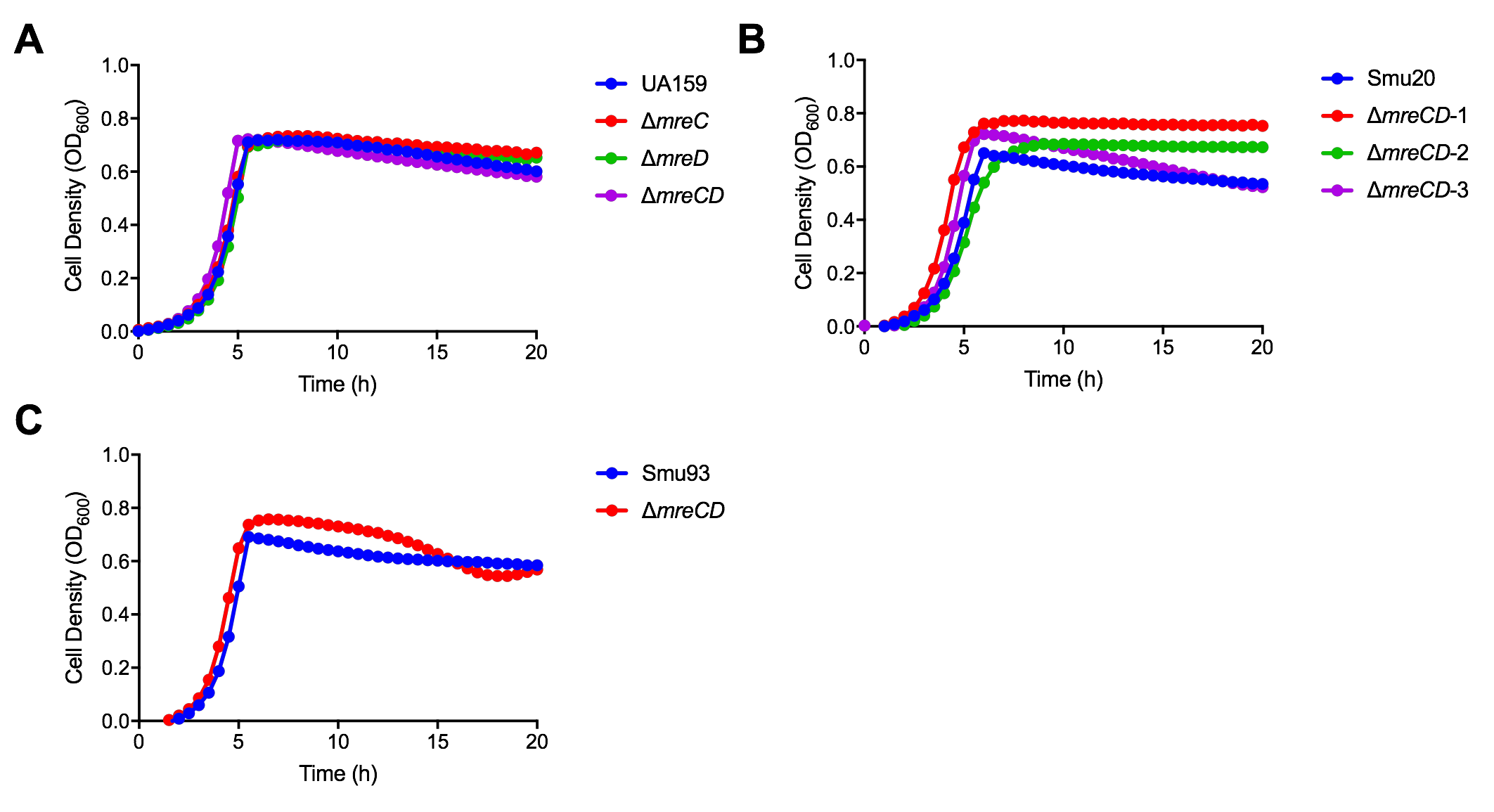
**

**Figure S2. Growth curve analysis of ∆*mreCD* strains cultured in rich medium.** Wild-type and mutant derivatives were grown in brain heart infusion (BHI) broth with a sterile mineral overlay (for anaerobic conditions) at 37°C and growth was measured using a Bioscreen C automated growth curve instrument for 20 h.

**Smu20_03743**

**Selected features:**

5-203: Predicted hydrolase (HAD superfamily)

**Nonsynonymous mutation:** N261K

mitaivfdvddtiydqqapyriavgkcfpdfdmaninqayirfrhysdtgfprvmanewtteyfrfwrcqktlldfdyrqigeeegqyfqeiyekeldqismldemrltldflkeknipmgvitngptehqlkkvkklglydyvdpkrvlvsqatgfqkpqkeifnlaaeqfemnpdttlyvgdsydndikgahdsgwhsmwfnhrgrrlkagtkpvfdlqidsfeqlygavkvlfdlpshkfifdandytnpilqlgin**n**glmmaaerllesnisidkvvillrldknqerilrlkyan

**Smu20_06741**

**Selected features:**

1-114: CheY-homologous receiver domain

146-243: LytTR DNA-binding domain

**Nonsynonymous mutation:** S59L

MNILILDDEMLARQELTFLIQQSKELDHPDIFEAEDISSAEKILFRQQIDLIFLDISL**S**EENGFTLANQLEQLAHPPLVVFATAYDHYAVKAFESNAADYILKPFEQERVDKALAKVKKIQHLSTIDETATTEKKGMELLTLTLADRSIVLKMPDIVAASIEDGELTVSTKNTSYTIKKTLNWFKTRAKTNYFLQIHRNTVVNLEMIQEIQPWFNHTLLLVMVNGEKFPVGRSYMKELNAHLTL

**Figure S3. Nonsynonymous mutations introduced during *mreCD* deletion in *S. mutans* strain Smu20.** Two single-nucleotide polymorphisms (SNPs) were observed in the genome of ∆*mreCD*_Smu20_. These were within gene Smu20_03743 (AAT>AAG; N261K) and gene Smu20_06741 (AGC>ATC; S59L). The nonsynonymous mutations are highlighted in red within the two protein amino acid sequences.

**Table S1 Quantitative proteomics comparing wild-type and *∆mreCD S. mutans***

|  |  | **MreCD** | | | | | **UA159** | | | | |  |  |  |
| --- | --- | --- | --- | --- | --- | --- | --- | --- | --- | --- | --- | --- | --- | --- |
| **Gene symbol** | **Description** | **1** | **2** | **3** | **4** | **5** | **1** | **2** | **3** | **4** | **5** | **logFC** | **P.Value** | **adj.P.Val** |
| **SMU_2134** | Transcriptional regulator | 23.3 | 23.3 | 23.3 | 23.3 | 23.4 | 21.4 | 21.5 | 21.2 | 21.3 | 21.2 | 2.02 | 9.26E-12 | 6.99E-09 |
| **SMU_198c** | Conjugative transposon protein | 20.3 | 20.3 | 20.5 | 20.4 | 20.2 | 19.4 | 19.4 | 19.3 | 19.2 | 19.0 | 1.08 | 1.10E-07 | 8.65E-06 |
| **SMU_1641c** | CsbD-like domain-containing protein | 24.8 | 24.9 | 25.3 | 25.3 | 25.4 | 24.2 | 24.1 | 24.2 | 23.9 | 24.2 | 1.03 | 4.51E-06 | 8.65E-05 |
| **SMU_1945** | VTC domain-containing protein | 21.5 | 21.5 | 21.5 | 21.6 | 21.3 | 22.7 | 22.6 | 22.4 | 22.4 | 22.5 | -1.01 | 1.61E-08 | 2.70E-06 |
| **bacA1** | Bacitracin synthetase 1 BacA | 25.6 | 25.2 | 25.1 | 25.7 | 25.0 | 26.5 | 26.6 | 26.4 | 26.1 | 26.3 | -1.05 | 2.19E-05 | 2.44E-04 |
| **SMU_1341c** | Gramicidin S synthetase | 24.8 | 24.4 | 24.2 | 24.9 | 24.1 | 25.7 | 25.8 | 25.5 | 25.3 | 25.4 | -1.07 | 3.77E-05 | 3.63E-04 |
| **gtfC** | Glucosyltransferase-SI | 22.8 | 22.8 | 22.7 | 22.6 | 22.6 | 23.9 | 23.9 | 23.4 | 23.9 | 23.9 | -1.10 | 6.26E-08 | 6.89E-06 |
| **SMU_219** | IrrE N-terminal-like domain-containing protein | 16.8 | 16.9 | 16.6 | 16.6 | 16.8 | 17.7 | 17.9 | 17.8 | 18.1 | 17.8 | -1.10 | 4.28E-08 | 5.82E-06 |
| **SMU_218** | Transcriptional regulator | 21.5 | 21.5 | 21.6 | 21.7 | 21.8 | 22.7 | 22.7 | 22.8 | 22.7 | 22.8 | -1.13 | 7.58E-10 | 2.86E-07 |
| **gtfB** | Glucosyltransferase-I | 24.5 | 24.7 | 24.6 | 24.4 | 24.7 | 26.1 | 25.9 | 25.5 | 26.1 | 26.0 | -1.32 | 1.58E-07 | 9.96E-06 |
| **mleS** | Malolactic enzyme | 22.1 | 22.3 | 22.3 | 22.2 | 22.4 | 23.6 | 23.6 | 23.7 | 23.6 | 23.7 | -1.40 | 1.18E-10 | 5.92E-08 |
| **SMU_2147c** | LysM domain-containing protein | 18.6 | 18.9 | 18.2 | 18.7 | 18.7 | 20.2 | 20.0 | 19.8 | 20.1 | 20.0 | -1.42 | 1.16E-07 | 8.65E-06 |
| **mreC** | Cell shape-determining protein MreC | 17.1 | 17.3 | 17.0 | 17.3 | 17.8 | 22.6 | 22.6 | 22.6 | 22.6 | 22.6 | -5.31 | 2.47E-13 | 3.73E-10 |

**Table S2 RNAseq reads in wild-type and *∆mreCD S. mutans***

| **Gene ID** | **Name** | **Product** | **1_UA159_BHI** | **2_UA159_BHI** | **3_UA159_BHI** | **4_mreCD_BHI** | **5_mreCD_BHI** | **6_mreCD_BHI** | **log2 fold change** | **FDR** |
| --- | --- | --- | --- | --- | --- | --- | --- | --- | --- | --- |
| **SMU_1908c** | NA | hypothetical protein | 1958 | 1963 | 2212 | 204 | 604 | 349 | -2.498656753 | 6.61E-09 |
| **SMU_1912c** | NA | hypothetical protein | 2337 | 2206 | 2120 | 237 | 645 | 385 | -2.469541812 | 7.56E-13 |
| **SMU_1910c** | NA | hypothetical protein | 5111 | 4980 | 5628 | 672 | 1760 | 930 | -2.30557251 | 1.24E-12 |
| **SMU_1913c** | NA | hypothetical protein; immunity protein, BLpL-like | 5752 | 5579 | 6214 | 784 | 1944 | 1092 | -2.269427169 | 1.32E-12 |
| **SMU_152** | NA | hypothetical protein | 1011 | 922 | 1022 | 133 | 314 | 194 | -2.262639166 | 3.16E-10 |
| **SMU_1903c** | NA | hypothetical protein | 779 | 620 | 725 | 95 | 266 | 142 | -2.165819136 | 6.28E-09 |
| **SMU_1909c** | NA | hypothetical protein | 6155 | 7223 | 8528 | 1033 | 2694 | 1471 | -2.154301893 | 1.50E-12 |
| **SMU_1914c** | bip cipB | bacteriocin protein, BlpO-like | 4670 | 6622 | 7918 | 865 | 2451 | 1296 | -2.149635038 | 5.76E-10 |
| **SMU_423** | nlmD | possible bacteriocin | 498 | 663 | 789 | 91 | 232 | 140 | -2.141816723 | 1.53E-08 |
| **SMU_1904c** | NA | hypothetical protein | 5090 | 4888 | 5717 | 870 | 1916 | 1030 | -2.095316225 | 5.88E-11 |
| **SMU_1905c** | NA | hypothetical protein | 1009 | 939 | 1043 | 172 | 373 | 207 | -2.041000608 | 2.45E-09 |
| **SMU_153** | NA | hypothetical protein | 478 | 340 | 402 | 60 | 151 | 105 | -2.010304273 | 2.15E-10 |
| **SMU_151** | nlmB | non-lantibiotic mutacin IV B | 780 | 806 | 859 | 141 | 317 | 210 | -1.918503925 | 1.52E-08 |
| **SMU_150** | nlmA | non-lantibiotic mutacin IV A | 521 | 654 | 774 | 142 | 277 | 138 | -1.842222557 | 8.57E-08 |
| **SMU_1906c** | NA | bacteriocin-related protein | 3203 | 4370 | 5294 | 751 | 2016 | 1042 | -1.841212589 | 1.02E-07 |
| **SMU_211c** | NA | hypothetical protein | 32 | 58 | 33 | 12 | 15 | 8 | -1.800284382 | 2.57E-07 |
| **SMU_201c** | NA | conserved hypothetical protein | 125 | 143 | 143 | 23 | 60 | 45 | -1.716790768 | 4.43E-14 |
| **SMU_204c** | NA | hypothetical protein | 40 | 46 | 45 | 8 | 19 | 15 | -1.658114185 | 5.35E-07 |
| **SMU_209c** | NA | hypothetical protein | 73 | 85 | 80 | 14 | 36 | 27 | -1.653228873 | 9.12E-11 |
| **SMU_1984** | cglC comGC comYC | competence protein ComYC | 48 | 72 | 22 | 13 | 15 | 19 | -1.559616151 | 7.10428E-06 |
| **SMU_197c** | NA | hypothetical protein | 295 | 290 | 306 | 67 | 150 | 94 | -1.557573415 | 2.94E-17 |
| **SMU_202c** | NA | conserved hypothetical protein/Streptococcus-specific protein | 99 | 124 | 111 | 24 | 57 | 35 | -1.554400658 | 7.10E-12 |
| **SMU_200c** | NA | hypothetical protein | 30 | 41 | 43 | 13 | 15 | 11 | -1.549226144 | 1.29327E-06 |
| **SMU_194c** | NA | conserved hypothetical protein, phage-related | 26 | 21 | 19 | 7 | 13 | 3 | -1.533249482 | 0.000792244 |
| **SMU_1981c** | comG comGF | competence protein G | 61 | 77 | 46 | 14 | 26 | 24 | -1.523100385 | 1.04656E-06 |
| **SMU_206c** | NA | hypothetical protein | 50 | 44 | 44 | 13 | 28 | 8 | -1.522070954 | 1.04656E-06 |
| **SMU_212c** | NA | hypothetical protein | 19 | 17 | 21 | 5 | 11 | 4 | -1.520192046 | 0.000746695 |
| **SMU_199c** | NA | hypothetical protein | 54 | 58 | 42 | 12 | 23 | 19 | -1.519257447 | 4.52514E-06 |
| **SMU_191c** | NA | phage-related integrase | 72 | 52 | 64 | 10 | 33 | 26 | -1.485100686 | 1.43E-07 |
| **SMU_205c** | NA | conserved hypothetical protein | 106 | 132 | 134 | 34 | 62 | 42 | -1.448148829 | 7.10E-12 |
| **SMU_1982c** | NA | conserved hypothetical protein | 89 | 85 | 38 | 23 | 31 | 24 | -1.425004895 | 2.08882E-06 |
| **SMU_198c** | tpn | conjugative transposon protein | 292 | 304 | 283 | 65 | 158 | 120 | -1.393613027 | 1.42E-15 |
| **SMU_626** | celB comEC | competence protein; possible integral membrane protein | 267 | 378 | 172 | 71 | 139 | 107 | -1.361074641 | 5.31E-08 |
| **SMU_196c** | NA | immunogenic secreted protein (transfer protein) | 112 | 158 | 156 | 32 | 85 | 54 | -1.358607694 | 1.28E-09 |
| **SMU_1896c** | NA | hypothetical protein | 1054 | 1535 | 1283 | 336 | 724 | 485 | -1.354471408 | 4.72E-10 |
| **SMU_574c** | lrg | effector of murein hydrolase | 90 | 904 | 824 | 113 | 491 | 187 | -1.340358756 | 0.092254622 |
| **SMU_1983** | cglD comGD comYD | competence protein ComYD | 73 | 127 | 45 | 25 | 36 | 35 | -1.317014228 | 1.75409E-05 |
| **SMU_1980c** | NA | conserved hypothetical protein | 122 | 139 | 69 | 42 | 54 | 35 | -1.30624594 | 6.99E-07 |
| **SMU_498** | comFA | late competence protein F | 87 | 133 | 69 | 21 | 58 | 43 | -1.267725059 | 1.1273E-05 |
| **SMU_1985** | cglB comGB comYB | competence protein; general (type II) secretory pathway protein | 196 | 252 | 140 | 60 | 95 | 88 | -1.254921054 | 8.57E-08 |
| **SMU_625** | celA comEA | competence protein | 145 | 207 | 100 | 50 | 69 | 66 | -1.252308261 | 1.24813E-06 |
| **SMU_1895c** | NA | hypothetical protein | 951 | 1034 | 833 | 301 | 539 | 361 | -1.236659392 | 1.50E-12 |
| **SMU_1987** | cglA comGA comYA | late competence protein; type II secretion system protein E | 233 | 316 | 190 | 77 | 126 | 109 | -1.228940684 | 8.64E-09 |
| **SMU_1001** | dprA | DNA processing protein, Smf family | 51 | 97 | 35 | 23 | 31 | 24 | -1.199086242 | 0.00022078 |
| **SMU_207c** | NA | transcriptional regulator | 129 | 143 | 128 | 44 | 76 | 61 | -1.152950068 | 5.11E-08 |
| **SMU_208c** | NA | conserved hypothetical protein, FtsK/SpoIIIE family | 247 | 280 | 294 | 81 | 168 | 135 | -1.122365944 | 6.05E-09 |
| **SMU_2147c** | NA | conserved hypothetical protein | 12295 | 15338 | 11793 | 3733 | 9536 | 6382 | -1.057599205 | 2.71E-09 |
| **SMU_216c** | NA | hypothetical protein | 41 | 46 | 42 | 15 | 31 | 19 | -1.008810685 | 0.000790982 |
| **SMU_883** | dexB | glucan 1,6-alpha-glucosidase | 678 | 746 | 867 | 1216 | 2499 | 1096 | 1.023373169 | 7.41E-08 |
| **SMU_1565** | malM | 4-alpha-glucanotransferase | 2747 | 2763 | 3533 | 4615 | 7809 | 6173 | 1.03183305 | 5.42E-10 |
| **SMU_2037** | dexS treC | trehalose-6-phosphate hydrolase | 7551 | 8076 | 12912 | 18194 | 30211 | 12189 | 1.064401488 | 0.000228955 |
| **SMU_2038** | pttB treB | phosphotransferase system, trehalose-specific IIBC component (EIIBC-tre) | 8392 | 8527 | 13603 | 19362 | 29277 | 15517 | 1.06980865 | 1.96731E-05 |
| **SMU_1571** | msmK | ABC-type transport system ATP-binding protein (maltose) | 1080 | 996 | 1390 | 1873 | 3958 | 1921 | 1.104442653 | 1.10E-09 |
| **SMU_877** | aga | alpha-galactosidase (melibiase) | 487 | 441 | 834 | 1080 | 1934 | 890 | 1.107545768 | 1.92027E-05 |
| **SMU_882** | msmK | multiple sugar-binding transport ATP-binding protein MsmK | 447 | 545 | 613 | 936 | 1864 | 763 | 1.10936745 | 3.22E-07 |
| **SMU_1568** | malE | maltose / maltodextrin-binding protein | 3710 | 3357 | 5013 | 6645 | 12894 | 7369 | 1.113226441 | 1.28E-09 |
| **SMU_182** | sloA | ABC transporter, ATP-binding protein, iron and/or manganese | 592 | 686 | 469 | 904 | 1723 | 1176 | 1.113658889 | 7.10E-12 |
| **SMU_1569** | malC malF | maltodextrin ABC transport system permease | 945 | 769 | 1155 | 1637 | 3075 | 1684 | 1.118710015 | 1.48E-09 |
| **SMU_1570** | malG malX | maltose / maltodextrin ABC transport system (permease) | 576 | 544 | 681 | 990 | 2040 | 1054 | 1.134432349 | 7.10E-12 |
| **SMU_179** | NA | conserved hypothetical protein (possible oxidoreductase) | 199 | 201 | 494 | 531 | 1262 | 540 | 1.285432035 | 0.0002134 |

**Table S3 Strains and plasmids used in this study**

| **Strain** | **Description** | **Source** |
| --- | --- | --- |
| *S. mutans* strains |  |  |
| UA159 | Wild-type | Burne Lab |
| Smu20 | Wild-type, clinical isolate |  |
| Smu93 | Wild-type, clinical isolate |  |
| *∆mreC* | *mreC*::*aphA3*, kanamycin resistant | This study |
| *∆mreD* | *mreD*::*aphA3*, kanamycin resistant | This study |
| *∆mreCD* | *mreCD*::*aphA3*, kanamycin resistant | This study |
| *∆mreCD*_Smu20_ | *mreCD*::*aphA3*, kanamycin resistant | This study |
| *∆mreCD*_Smu93_ | *mreCD*::*aphA3*, kanamycin resistant | This study |
| *S. gordonii* DL1 | Wild-type | Burne lab |
| *S. sanguinis* SK150 | Wild-type | Burne lab |
| *E. coli* strains |  |  |
| 10-beta | Cloning host, derivative of DH10B | New England Biolabs |
| BTH101 | Reporter strain for BACTH assay | Euromedex |
| Plasmids |  |  |
| pALH124 |  |  |
| pKT25 | BACTH vector, ampicillin resistance | Euromedex |
| pUT18C | BACTH vector, kanamycin resistance | Euromedex |

**Table S4 Oligonucleotide primers used in this study**

| **Oligonucleotide name** | **Sequence (5’ – 3’)** | **Description** |
| --- | --- | --- |
| B2H-mreC-FW | ATGCTCTAGAGATGAGATTGTCACGTTTTA | B2H cloning |
| B2H-mreC-RW | ATGCGGTACCCGATCACCTACTAAAGTTACA | B2H cloning |
| B2H-mreD-FW | ATGCTCTAGAGATGTCTATCTTTAAAAATAAATTATTT | B2H cloning |
| B2H-mreD-RW | ATGCGGTACCCGTAGGTAAATCCTTTCTAAAAGTTT | B2H cloning |
| B2H-pbp2b-FW | ATGCTCTAGAGATGGTATCCCAAAAAAATAAATC | B2H cloning |
| B2H-pbp2b-RW | ATGCGGTACCCGCTGATTCATTGGATACATCTT | B2H cloning |
| B2H-rodA-FW | ATGCTCTAGAGATGGCAAGCAAAAAAAAG | B2H cloning |
| B2H-rodA-RW | ATGCGGTACCCGACTTGTAAGACTCTCCTT | B2H cloning |
| B2H-rodZ-FW | ATGCTCTAGAGATGAACAAGATAGGTGATAC | B2H cloning |
| B2H-rodZ-RW | ATGCGGTACCCGTTGGACTGTCAAATTAATATTAGT | B2H cloning |
| B2H-pbp1a-FW | ATGCTCTAGAGATGACAGTTCTAAAATATGGA | B2H cloning |
| B2H-pbp1a-RV25 | ATGCGAATTCTTATGGATTAGAAGATGTCGTA | B2H cloning |
| B2H-pbp1a-RV18C | ATGCGAATTCCGTGGATTAGAAGATGTCGTA | B2H cloning |
| pKT25seqF | GTCAAGGTGATCGGCAATG | B2H sequencing |
| pKT25seqR | ggcctcttcgctattacgc | B2H sequencing |
| pUT18CseqF | AGTTCTCGCCGGATGTACTG | B2H sequencing |
| pUT18CseqR | ggtgatgacggtgaaaacct | B2H sequencing |
| SMu.20A | tagacatatcgcgggatgga | *mreC* cloning |
| SMu.20BKm | ATGCGGATCCCCGTGATAACAAAAACAATAAAACG | *mreC* cloning |
| SMu.20CKm | ATGCGGATCCTGTTGAAGGTGTGTCTGTTGG | *mreC* cloning |
| SMu.20D | gccagtatattaaaaattaaggaagg | *mreC* cloning |
| SMu.20E | ctgaagggaacggtcttgaa | *mreC* sequencing |
| SMu.20F | ctgcgcatcaaagtcatcag | *mreC* sequencing |
| SMu.21A | ccccggtttcttggttagat | *mreD* cloning |
| SMu.21BKm | ATGCGGATCCAAGAAAGGCAAATAAAATGACAAA | *mreD* cloning |
| SMu.21CKm | ATGCGGATCCTGGCTTTTCTCTTCTTTCAAAAAC | *mreD* cloning |
| SMu.21D | gctggtagctgcgttactttt | *mreD* cloning |
| SMu.21E | agcgctgatgagatcgtaca | *mreD* sequencing |
| SMu.21F | gctgcttgattttcttgttgc | *mreD* sequencing |
| Smu20_03743F | GGCTGGCATTCTATGTGGTT | SNP sequencing |
| Smu20_03743R | ttcagccttcaacccaaatc | SNP sequencing |
| Smu20_06741F | tacagatcaaggaacaaccgtct | SNP sequencing |
| Smu20_06741R | GCCAAAGCTTTATCCACACG | SNP sequencing |
